# Multiple logistic regression analysis of risk factors in elderly pneumonia patients: QTc interval prolongation as a prognostic factor

**DOI:** 10.1186/2049-6958-9-59

**Published:** 2014-11-22

**Authors:** Yasuyuki Taooka, Gen Takezawa, Miki Ohe, Akihisa Sutani, Takeshi Isobe

**Affiliations:** Department of General Medicine, Aki-Ohta Hospital, Shimodono-Gohchi 236, Aki-Ohta-Cho, Yamagata-Gun, Hiroshima, 731-3622 Japan; Division of Clinical Oncology and Respiratory Medicine, Department of Internal Medicine, Shimane University Faculty of Medicine, Izumo, Japan

**Keywords:** Elderly, Electrocardiogram, QTc interval, Pneumonia

## Abstract

**Background:**

Acute pneumonia is a serious problem in the elderly and various risk factors have already been reported, but the involvement of QTc interval prolongation remains uncertain. The aim of this study was to elucidate the prognostic factors for the development of pneumonia in elderly patients and to study the possible involvement of QTc interval prolongation.

**Methods:**

The subjects were 249 hospitalized pneumonia patients more than 65 years old in Aki-Ohta Hospital from January 2010 to December 2013. Community-acquired pneumonia patients and nursing care and healthcare-associated pneumonia patients were included in the study. The pneumonia severity index, vital signs, blood chemistry data and ECG findings were retrospectively compared using multiple logistic regression analysis.

**Results:**

39 patients died within 30 days from onset. The clinical features related to poor prognosis were: advanced age, past history of cerebral vascular disease and/or diabetes mellitus, decreased serum albumin level, higher CURB-65 or PORT index scores and QTc interval prolongation. Patients showing a prolonged QTc interval had a higher mortality than those with a normal QTc interval. A prolonged QTc interval was not related to serum calcium concentration and/or treatment with QTc prolongation drug, clarithromycin or azithromycin, but related to age, lower albumin concentration and past history of diabetes mellitus.

**Conclusions:**

These findings suggest potential prognostic factors for pneumonia in elderly patients, including a prolonged QTc interval (> 0.44 seconds).

## Background

Although pneumonia is a common disease, the mortality of elderly pneumonia patients still remains high [1, 2]. As classifications of the severity of pneumonia, CURB-65 [3] and the PORT-study pulmonary severity index (PSI) [4] show good correlations with pneumonia mortality and they are well utilized. Unlike younger patients, elderly patients usually have several other diseases at the time of onset of pneumonia [5–7], since the prognosis of their pneumonia is affected by their concomitant diseases, and it is possible that pneumonia in the elderly has various and different risk factors than pneumonia in younger patients. Therefore, we thought that it would be appropriate to analyze risk factors for pneumonia other than the CURB-65 and PORT-study PSI in elderly patients. Through daily clinical work at the bedside, we came to know that the electrocardiogram (ECG) QTc interval was prolonged in some elderly pneumonia patients whose prognosis was not good. At the beginning, we thought that it was related to medications they were given, but we could not find any adequate explanations for the QTc interval prolongation in many cases.

Physiologically, QTc interval prolongation reflects prolonged total duration of ventricular myocardial repolarization. It has been reported that not only hypocalcaemia and medication, but also heart failure and ischemic heart disease are involved in QTc interval prolongation [8, 9]. Furthermore, a prolonged QTc interval was recently found to increase the rates of all causes of death [10]. In respiratory disease, mortality of COPD patients is associated with QTc interval [11]. However, it is still uncertain whether a prolonged QTc interval is a risk factor for a poor outcome in pneumonia patients. We speculated it was possible that pneumonia induced-hypoxemia, secondary pulmonary hypertension, or heart failure might affect QTc interval prolongation.

The hypothesis of this study was that a prolonged QTc interval might be associated with a poor prognosis in elderly pneumonia patients. Therefore, the mortality rates of elderly pneumonia patients in our hospital were analyzed.

## Methods

### Study design

The study was conducted in the 199-bed, Aki-Ohta Hospital (Hiroshima, Japan), which shared primary care- and critical care-medicine. The subjects were 249 hospitalized pneumonia patients (132 males, 117 females; mean age 79.62 ± 8.67 years) from January 2010 to December 2013. All consecutively hospitalized patients more than 65 years old were included, with 178 community-acquired pneumonia (CAP) patients and 71 nursing care and health care-associated pneumonia (NHCAP) patients. The Japan Respiratory Society Guidelines documented the new category, NHCAP [12], which includes patients with any of the following: residence in a long-term nursing home setting or healthcare home; discharge from hospital during the preceding 90 days; elderly persons or physically disabled persons who need healthcare; persons continuously receiving endovascular therapy in an ambulatory setting. Pneumonia was defined as the presence of symptoms of a lower respiratory tract infection with new infiltrates on a chest X-ray and the absence of an alternative diagnosis. The attending physician decided on hospital admission and antibiotic treatment. The research team followed all patients, recording clinical and laboratory data. At the time of diagnosis, the PSI, vital signs, blood chemistry data, and ECG findings were recorded, and they were retrospectively compared using multiple logistic regression analysis; the variables included serum C-reactive protein (CRP), white blood cell count (WBC) and the serum albumin concentration. The severity of pneumonia was graded by the CURB-65 score (confusion, uremia, respiratory rate, low blood pressure, age 65 years or greater) [3]. PORT-study points were summed and also used as the PSI [4]. These data were compared using multiple logistic regression analysis. Patients were followed up until discharge from the hospital or death. The study was approved by the institutional review board of Shimane University, School of Medicine (approval number: 437-21-01-20) and the local ethics committee of Aki-Ohta Hospital, and all subjects gave written informed consent for participation in the study.

### Endpoint of the study

Patients were followed until discharge from the hospital or death and those who died within 30 days from admission constituted the fatal outcome group (30-day nonsurvivors).

### Recording QT intervals

QT intervals were measured on a resting ECG tracing in lead II. The QT interval was measured manually from the starting point of the QRS complex to the terminal point of the down slope of the T wave. QTc was calculated according to Bazett’s formula [13]: QTc* =* QT*/*(RR)^1*/*2^. A QTc interval *>*0.44 seconds was considered abnormally prolonged [8]. No QTc measurement was obtained in the 13 patient (5 patient who died and 8 patient who survived) in whom no adequate RR interval was available.

### Statistical analysis

Statistical analysis was performed using computer software (Excel Statistics 2012, SSRI Co., Ltd., Tokyo, Japan). All data are expressed as means ± standard deviation (S.D.). The chi-square test, Fisher’s exact test and the Mann-Whitney U test were used to evaluate the differences in the clinical features of pneumonia patients. Using multiple logistic regression analysis, the patients’ relative risks were calculated. When the 95% confidence interval (95% CI) of the relative risk excluded a value of one, the risk was considered significant and a probability value of less than 0.05 was considered significant.

## Results and discussion

A total of 39 patients (23 males, 16 females; 84.37 ± 8.32 years old) died within 30 days (30-day nonsurvivors), and 210 patients (109 males, 101 females; mean age 78.20 ± 7.62 years) survived (30-day survivors), for a mortality rate of 15.7% (Table 1). Age, severity of pneumonia (CURB-65 and PSI), lower serum albumin and QTc interval prolongation were significantly different between the fatal outcome group and the surviving group. About the ratio of taking QTc prolongation drug, statin, clarithromycin or azithromycin, there were no differences between the groups (data not shown). Patients in the fatal outcome group had a higher rate of a past history of diabetes mellitus (DM) and cerebral vascular disease (CVD) (Table 2). Next, multiple logistic regression analysis of the clinical features of the pneumonia patients (30-day survivors group *versus* 30-day nonsurvivors group) was performed (Table 3). The odds ratios of age, CURB-65, PSI, lower albumin, QTc interval prolongation and past history of DM and CVD were significantly higher in the fatal outcome group. The odds ratio of QTc interval prolongation in the poor prognosis group (died within 30 days from admission) was 3.792 (95% CI = 1.100-13.071).

**Table 1:** **Univariate analysis of differences in clinical features in pneumonia patients (1)**

Factors	30-day survivors		30-day nonsurvivors		***p value***
	Mean ± S.D.	n	Mean ± S.D.	n	
**Mean age (years)**	78.20 ± 7.62	210	84.37 ± 8.32	39	< 0.0001
**CURB-65**	2.01 ± 0.71	210	3.56 ± 0.72	39	< 0.0001
**PSI**	85.10 ± 22.92	210	117.08 ± 19.16	39	< 0.0001
**WBC (/mm** ^**3**^ **)**	10,203.8 ± 4,409.3	210	10,053.6 ± 5,441.5	39	0.4986
**CRP (mg/dL)**	8.57 ± 6.14	210	9.12 ± 6.07	39	0.6169
**Albumin (g/dL)**	3.53 ± 0.52	210	2.87 ± 0.57	39	< 0.0001
**HR (beat/minute)**	85.09 ± 13.13	210	94.23 ± 21.22	39	0.0541
**QTc (seconds)**	0.424 ± 0.032	202	0.456 ± 0.007	34	< 0.0001

Data represent the numbers of patients. CRP, C-reactive protein; PSI, pneumonia severity index; WBC, white blood cell count. All data are expressed as means ± standard deviation of the means. Counting points of CURB-65 and PORT-study as the severity of pneumonia are cited from reference [3] and [4].

**Table 2:** **Univariate analysis of differences in clinical features in pneumonia patients (2)**

Factors	Categories	30-day survivors	30-day nonsurvivors	***p value***
**Gender**	Male	109	23	0.5237
	Female	101	16	
**Past history of**				
**Heart failure**	+	38	9	0.6119
	-	172	30	
**COPD**	+	50	7	0.5535
	-	160	32	
**CVD**	+	66	22	0.0049
	-	144	17	
**DM**	+	19	12	0.0004
	-	191	27	
**Dementia**	+	62	16	0.2171
	-	148	23	

Data represent the numbers of patients. COPD, chronic obstructive lung disease; CVD, cerebral vascular disease; DM, diabetes mellitus.

**Table 3:** **Multiple logistic regression analysis of differences in clinical features in pneumonia patients**

Risk factors	Categories		Odds ratio		(95% CI)	***p value***
			30-day survivors	30-day nonsurvivors		
**Mean age (years)**	≥ 80	< 80	1	2.789	(1.319 - 5.895)	0.0072
**CURB-65**	≥ 3	< 3	1	13.106	(5.150 - 33.357)	< 0.0001
**PSI**	≥ 131	< 131	1	6.444	(3.031 - 13.702)	< 0.0001
**Albumin (g/dL)**	< 3.0	≥ 3.0	1	9.609	(2.036 - 45.354)	0.0043
**QTc (seconds)**	> 0.44	≤ 0.44	1	3.792	(1.100 - 13.071)	0.0348
**Past history of**						
**CVD**	+	-	1	4.217	(1.173 - 15.165)	0.0275
**DM**	+	-	1	4.267	(1.800 - 10.116)	0.0010

Data represent the numbers of patients. PSI, pneumonia severity index. Counting points of CURB-65 and PORT-study as the severity of pneumonia are cited from reference [3] and [4]. CVD, cerebral vascular disease; DM, diabetes mellitus. 95% CI: 95% confidence interval.

These parameters were then compared between two groups: QTc> 0.44 seconds and QTc ≤ 0.44 seconds**.** Age, CURB-65, higher CRP and lower albumin showed significant differences (Table 4). In cases with low serum albumin, serum calcium concentration was adjusted to avoid the effect of lower albumin as following; corrected serum calcium concentration (mg/dl) = (concentration of serum calcium) (mg/dl) + [4 – (serum albumin concentration) (g/dl)] [14]. Corrected serum calcium concentration showed no significant difference between the groups. The prognosis and a past history of DM also showed significant differences (Table 5). Multiple logistic regression analysis of clinical features in pneumonia patients (QTc> 0.44 versus QTc ≤ 0.44) are shown in Table 6. The odds ratios were significantly higher for older age, poor prognosis, lower albumin and past history of DM in the QTc interval prolongation group. Odds ratio of a poor prognosis (dead within 30 days from admission) was 4.145 (95 % CI = 1.849-9.291).

**Table 4:** **Univariate analysis of differences in clinical features in pneumonia patients with/without QTc prolongation (1)**

Factors	QTc > 0.44		QTc ≤ 0.44		***p value***
	Mean ± S.D.	n	Mean ± S.D.	n	
**Mean age (years)**	82.58 ± 7.93	74	79.86 ± 7.51	162	0.0128
**CURB-65**	2.49 ± 0.99	74	2.18 ± 0.85	162	0.0414
**PSI**	112.71 ± 22.23	74	109.10 ± 21.50	162	0.3231
**WBC (/mm3)**	10,678.1 ± 4,806.3	74	10,060.2 ± 4,610.5	162	0.4693
**CRP (mg/dL)**	10.83 ± 7.05	74	8.00 ± 5.83	162	0.0048
**Albumin (g/dL)**	3.22 ± 0.56	74	3.50 ± 0.57	162	0.0024
**Corrected Ca (mg/dL)**	8.82 ± 0.58	61	8.67 ± 0.61	138	0.2376
**HR (beat/minute)**	87.76 ± 13.81	74	86.03 ± 14.78	162	0.4980
**QTc (seconds)**	0.463 ± 0.020	74	0.412 ± 0.024	162	< 0.0001

Data represent the numbers of patients. CRP, C-reactive protein; PSI, pneumonia severity index; WBC, white blood cell count. All data are expressed as means ± standard deviation of the means. Counting points of CURB-65 and PORT-study as the severity of pneumonia are cited from reference [3] and [4]. Corrected serum calcium concentration is cited from reference [14].

**Table 5:** **Univariate analysis of differences in clinical features in pneumonia patients with/without QTc prolongation (2)**

Factors	Categories	QTc > 0.44	QTc ≤ 0.44	***p value***
**Gender**	Male	32	88	0.1374
	Female	42	74	
**Prognosis**	Nonsurvivors	23	11	p < 0.0001
**(30-day)**	Survivors	51	151	
**Past history of**				
**Heart failure**	+	16	27	0.4635
	-	58	135	
**COPD**	+	12	44	0.0952
	-	62	118	
**CVD**	+	31	55	0.3029
	-	43	107	
**DM**	+	17	13	0.0028
	-	57	149	
**Dementia**	+	23	50	0.9058
	-	51	112	

Data represent the numbers of patients. COPD, chronic obstructive lung disease; CVD, cerebral vascular disease; DM, diabetes mellitus.

**Table 6:** **Multiple logistic regression analysis of differences in clinical features in pneumonia patients with/without QTc prolongation**

Risk factors	Categories		Odds ratio		(95% CI)	***p value***
			QTc ≤ 0.44	QTc > 0.44		
**Age (years)**	≥ 80	< 80	1	2.3077	(1.242 - 4.286)	0.0081
**Prognosis**	Survivors	Nonsurvivors	1	4.145	(1.849 - 9.291)	0.0006
**(30-day)**						
**CURB-65**	≥ 3	< 3	1	3.289	(0.843 - 12.839)	0.0867
**CRP (mg/dL)**	≥ 10.	< 10.0	1	0.326	(0.173 - 2.595)	0.1493
**Albumin (g/dL)**	< 3.0	≥ 3.0	1	9.609	(2.036 - 45.354)	0.0043
**Past history of**						
**DM**	+	-	1	3.391	(1.328 - 8.66)	0.0110

Data represent the numbers of patients. CRP, C-reactive protein, Counting points of CURB-65 as the severity of pneumonia is cited from reference [3] and [4]. DM, diabetes mellitus. 95% CI, 95% confidence interval.

In the present study, the presence of QTc interval prolongation in pneumonia patients was found to correlate with a poor prognosis. As far as we know, this is the first report showing that QTc interval prolongation may be a predictive factor of increased mortality in pneumonia patients. Other than prolonged QTc interval, age [15], hypo-albuminemia [16], high CURB-65 [3, 17] or PORT-study PSI [4, 18] scores, past history of DM [19] and/or CVD [20] were associated with high mortality, compatible with previous reports as risk factors of pneumonia in elderly patients**.**

Although there has been a case report where high fever induced a prolonged QTc interval [21], there have been no reports describing that hypoxemia or infection prolonged the QTc interval. Therefore, it is necessary to find the cause of QTc interval prolongation in our cases and elucidate the contribution of multiple other factors**.** In our analysis, QTc prolongation was related with age, poor prognosis, lower albumin concentration and past history of DM. So, involvement of these factors as the cause of QTc interval prolongation was suspected.

In the present study the clinical background characteristics of elderly pneumonia patients are heterogeneous and show variety [22], and some but not all patients who died showed no changes in their QTc interval. However, there were several associated risk factors on logistic analysis. One was a past history of DM. There have been many recent papers on the QTc interval and DM [23–27]. Although the electrical and physiological mechanisms of QTc interval prolongation in DM are still uncertain, one possibility is that increased autonomic nervous system activity can be responsible for repolarization changes. The involvement of diabetic complications and the severity of DM have been found to be significantly associated with mortality and QTc interval prolongation [27]. Therefore, a past history of DM might be a candidate cause of QTc interval prolongation in our study.

CVD is also known as a cause of QTc prolongation [26–28]. In the present study, some patients, not a small proportion of the subjects, had a past history of CVD, which was compatible with previous reports [28–30].

Heart diseases such as ischemic heart disease and heart failure are associated with a higher rate of mortality and QTc interval prolongation [8–10]. An increased serum BNP concentration is also associated with QTc interval prolongation [28]. However, contrary to these previous reports, a past history of heart failure was not significantly associated with QTc interval prolongation in the present study. One reason for this is that atrial fibrillation cases were omitted, because it is impossible to measure the ECG QTc interval correctly in the presence of atrial fibrillation. It is not difficult to imagine that secondary acute heart failure would occur following acute respiratory failure in elderly pneumonia patients. Even if it occurs, because of acute pneumonia, secondary heart failure could become one cause of death [29]. Strictly, the present result came from an analysis of the relationship between a past history of heart failure and QTc interval prolongation and it was not about showing the existence of heart failure when pneumonia was diagnosed. Thus, the present result did not show that heart failure was not associated with prolongation of the QTc interval**.**

As a result of combination of these differences, QTc interval prolongation might occur. In this study it did not derive from the difference of the content of treatment with clarithromycin, or azithromycin. Since there were differences with past history between the fatal outcome and surviving groups, it was possible that the differences in content of medications other than clarithromycin and azithromycin might have been related to the cause of QTc interval prolongation [30]. But in the study, we could not detect the specific medication as the candidate of QTc prolongation. Therefore in this meaning, we supposed the differences in past history between the groups and their related increased autonomic nervous system activity might have been responsible for QTc interval changes. The important thing is the fact that the mortality rate of pneumonia patients who showed a prolonged QTc interval at the time of diagnosis was increased in this study.

One limitation of the present study was that the results were based on retrospective analysis. However, the subjects were all patients seen during the period of the study. Therefore, the possibility of having a bias would not be high. As already described, the clinical background characteristics of elderly pneumonia patients included multiple, various risk factors, including habits and community-based characteristics [15]. To show the involvement of QTc interval prolongation as a prognostic factor in elderly pneumonia, further prospective studies to evaluate the relevance of factors known to influence QTc intervals and factors potentially contributing to mortality in pneumonia will be necessary. Furthermore, since the present analysis did not distinguish between CAP and NHCAP cases, one can’t rule out the possibility that there were differences in risk factors between CAP and NHCAP cases [31]. In the present study, since the number of subjects was too small, such a comparison was not made and further studies will be necessary.

In clinical practice, QTc interval can be simply determined. Physicians should consider that QTc prolongation, besides advanced age, hypo-albuminemia, high CURB-65 and/or PORT-study PSI scores, past history of DM and/or CVD, might be useful factors in indentifying future risk of mortality and probable need for intense care in elderly pneumonia patients.

## Conclusion

In summary, the present study suggests several potential prognostic factors in elderly patients with pneumonia, including a prolonged QTc interval (> 0.44 seconds).

## Abbreviations

CAP: Community-acquired pneumonia
NHCAP: Nursing care and health care-associated pneumonia
CRP: C-reactive protein
PORT: Patient Outcome Research Team
ECG: Electrocardiogram
PSI: Pneumonia severity index
CVD: Cerebral vascular disease
COPD: Chronic obstructive pulmonary disease
DM: Diabetes mellitus
95% CI: 95% confidence interval.
